# Progressive Multifocal Encephalopathy Holmes Tremor Successfully Treated with Bilateral Deep Brain Stimulation

**DOI:** 10.5334/tohm.781

**Published:** 2023-09-01

**Authors:** Talita D. Rosa, Laura Dixon, Muhammad Ismail Khalid Yousaf, Victoria Holiday, Ajmal Zemmar, Joseph Neimat, Peter Hedera

**Affiliations:** 1 University of Louisville School of Medicine, Department of Neurology, US; 2 University of Louisville School of Medicine, Department of Neurosurgery, US

**Keywords:** Homes tremor, Rubral Tremor, Tremor, Deep brain Stimulation

## Abstract

**Background:**

We report a patient with bilateral HT treated with DBS.

**Case report:**

A 58-year-old man diagnosed with HIV/AIDS and progressive multifocal leukoencephalopathy (PML) presented with 20 years of bilateral arm tremor refractory to therapy. DBS was implanted on the left ventral intermediate nucleus and posterior subthalamic area (VIM/PSA). One year later, a right VIM/PSA DBS was implanted. At twelve months, there were no significant side-effects. With his DBS turned off and on, the Fahn-Tolosa-Marin scale was rated 82 and 58, respectively.

**Discussion:**

To our knowledge, this is the first report of bilateral DBS VIM/PSA treating HT with no significant side effects.

**Highlights:**

We report a successful treatment using deep brain stimulation of bilateral Holmes tremor that was caused by progressive multifocal encephalopathy. The patient achieved 30% improvement in tremor control with a meaningful improvement in his activities of daily living.

## Introduction

Holmes tremor (HT) is characterized by a large amplitude, irregular, lower frequency tremor that is present at rest and is worsened by action and posture, predominantly affecting the upper extremity [1,2]. HT has been reported as secondary to various conditions, including central nervous system trauma, tumors, infectious, vascular, and neurodegenerative causes. It can appear weeks to years after the causative lesion [3]. Although its precise pathology is yet unknown, it is suspected to involve the cerebello-thalamocortical or dentato-rubro-olivary pathways [2].

Medication management has been shown to improve HT resting tremor, with less robust response to postural and intention tremor [1]. However, medical therapy for patients with HT can be challenging and is seldom fully effective [3]. Medications that can be tried includes antiepileptic drugs (levetiracetam), levodopa, anticholinergics (trihexyphenidyl), and dopamine agonists. Even when effective, the effects of therapy sometimes can wean off over time.

More recently, a few cases of Holmes tremor were reported as having a good response to unilateral deep brain stimulation (DBS) in medically refractory tremors [4]. DBS has been shown to improve both resting and postural HT [1]. The most common target for DBS is the thalamic ventral intermediate nucleus (VIM), followed by the subthalamic nucleus (STN) and globus pallidus internus (GPi) [1,3]. Although there have been reports of dual stimulation targets, to our knowledge there are no cases of bilateral DBS reported in the literature.

We report a unique case of a patient with acquired Holmes tremor (HT) secondary to Human Immunodeficiency Virus (HIV) and progressive multifocal leukoencephalopathy (PML) effectively treated with bilateral deep brain stimulation (DBS).

## Case Description

A 58-year-old right-handed man presented with a 20-year history of bilateral arm tremor after a diagnosis of HIV/AIDS and progressive multifocal leukoencephalopathy (PML). At onset, he developed sudden gait disturbance with imbalance, visual disturbance, and right sided weakness. Previous imaging studies showed typical radiologic signs of PML (original images not available). His neurologic symptoms improved with anti-HIV therapy.

At the time of his first evaluation at our institution, his weakness, and visual changes had resolved. He presented with a four to five Hz moderate amplitude resting tremor of both arms that increased with posture and intention, especially with tasks such as writing and drinking. In addition, he presented with bradykinetic toe taps on the left, mildly bradykinetic bilateral finger taps more pronounced on the left, and bilateral finger to nose dysmetria.

**Figure 1:**
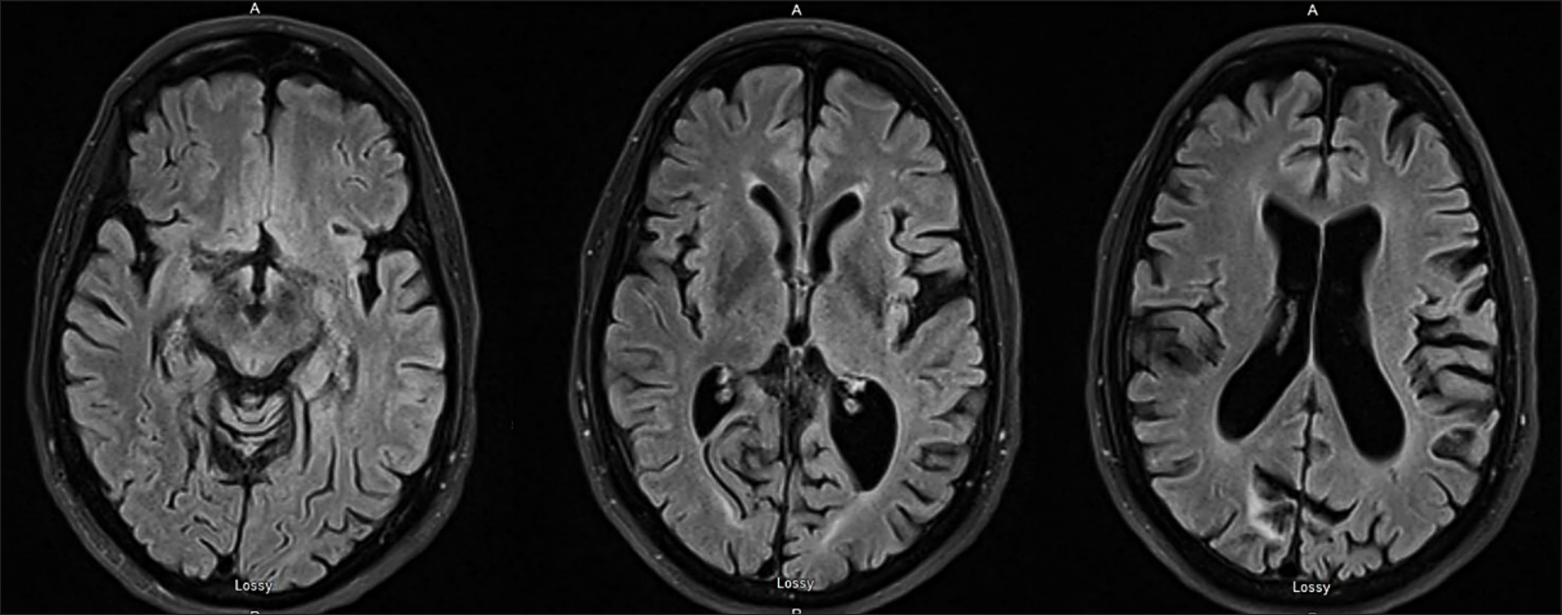
MRI brain. Preoperative MRI revealing late sequela of demyelination with brain atrophy and pathy areas of secondary axonal loss throughout the brain and concavity of the apex of the midbrain.

Follow up brain MRI showed areas of late sequela of demyelination with patchy areas of secondary axonal loss throughout the brain in a subcortical distribution and concavity of the apex of the midbrain with apparent sparing of the basal ganglia [Figure 1]. Propranolol at the highest dose 180 mg per day, topiramate at the highest dose 300 mg per day, and zonisamide at the highest dose 400 mg per day were tried without success. Carbidopa-levodopa at the maximum daily dose of 900 mg provided limited improvement that weaned off over time. Primidone was not tolerated even at the dose of 50 mg per day. Two years after he was first evaluated, his symptoms had not progressed but continued to cause significant functional impairment in activities of daily living (ADLs) such as writing, drinking, feeding, and preparing meals. After a preoperative evaluation that included discussion on DBS multidisciplinary board and a neuropsychology evaluation, he underwent DBS implantation (Abbott system) of the left ventral intermediate nucleus and posterior subthalamic area (VIM/PSA).

Although his right arm tremor was markedly improved with near complete resolution of his oscillatory right upper extremity tremor with continued dysmetria, his contralateral tremor was still resulting in significant disability. One year after, a repeat neuropsychology evaluation showed some decline in executive function, but he was still considered a good candidate for surgery. He underwent DBS implantation of his right VIM/PSA. At twelve months follow up, there were no significant side-effects.

The patient underwent neurosurgical DBS lead placement using the STarFix frame (FHC Inc. Bowdoin, ME, USA), coupled with microelectrode recordings and intraoperative testing to optimize contact placement (for details see Konrad et al. 2011). The Abbott Scientific Infinity 1.5 mm spaced directional DBS electrode and battery were placed in the VIM, planned according to standard stereotactic techniques. The right lead was placed one millimeter (mm) medial to the originally outlined target as intraoperative testing revealed capsular symptoms and two millimeters below to stimulate portions of the PSA (X: 14 mm lateral to midline, Y: one fourth of the anterior-posterior commissure (AC-PC) distance anterior to PC, Z: two millimeters below the level of the PC). The left lead had been placed 10 months prior at 15 mm lateral, three mm below and one fourth of the AC-PC distance anterior to the PC).

The final optimal DBS parameters in the left thalamus were monopolar stimulation (case positive), –2a,b, pulse width 60 msec, amplitude 4.0 mA, frequency 130 Hz, and in the right thalamus monopolar stimulation (case positive), –10a,b, pulse width 80 msec, amplitude 4.2 mA, and frequency 130 Hz.

**Figure 2:**
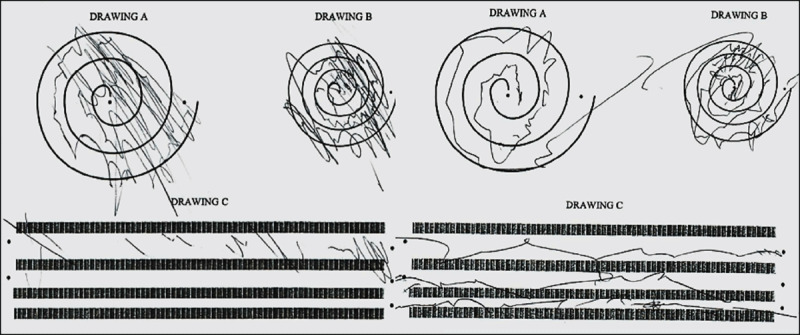
Fahn-Tolosa-Marin rating scale of tremor Drawings **A, B** and **C**. Drawings with stimulation on, using left hand (left) and right hand (right). Drawings with the stimulation off are not shown because the patient was not able to perform either task.

The Fahn-Tolosa-Marin (FTM) rating scale was obtained with the stimulation turned off, on, and on after setting adjustments (Table 1 A total score with the stimulation turned on without adjustments was not obtained because not all categories were measured. With his DBS stimulation turned off and on after setting adjustments, respectively, he scored 82 and 58 on the FTM rating scale [Table 1]. With the stimulation on, he was overall moderately tremulous on the right arm and was able to pour water with significant spilling (total score of nine). He was able to draw with the left hand with many errors and was not able to pour without spilling most of the water (total score 13). While the stimulation was off, he was unable to hold the pen to perform any of the FTM drawings [Figure 2] and was unable to complete the pouring task with either arm. His gait was ataxic at onset of symptoms and was unchanged after DBS, and on and off evaluation of his gait did not show any adverse effects of stimulation. He continued to require a cane to walk. The patient also reported improvements in his abilities to perform basic activities of daily living related to eating, drinking and hygiene.

**Table 1:** Fahn, Tolosa, Marin Tremor Rating scale with the deep brain stimulation turned on and off.


	OFF STIM			ON STIM			ON ADJUSTED STIM		
		
	REST	POST	ACTION	REST	POST	ACTION	REST	POST	ACTION

1. Face^1^	0	–	–	0	–	–	0	–	–

2. Tongue	0	0	–	0	0	–	0	0	–

3. Voice	–	–	1	–	–	1	–	–	1

4. Head	0	0	–	0	0	–	0	0	–

5. RUE	2	3	3	1	2	2	1	2	2

6. LUE	3	4	4	1	3	3	1	3	3

7. Trunk	0	0	–	0	0	–	0	0	–

8. RLE	0	0	0	0	0	0	0	0	0

9. LLE	0	0	0	0	0	0	0	0	0

Total	**5**	**7**	**8**	**––**	**–**	**–**	**2**	**5**	**6**

	**RIGHT**	**LEFT**	**N/A**	**RIGHT**	**LEFT**	**N/A**	**RIGHT**	**LEFT**	**N/A**

10. Handwriting^2^	–	–	4	–	–	–	–	–	3

11. Drawing A^3^	4	4	–	–	–	–	2	3	–

12. Drawing B	4	4	–	–	–	–	2	3	–

13. Drawing C	4	4	–	–	–	–	2	3	–

14. Pouring^4^	4	4	–	3	4	–	3	4	–

15. Speaking^5^	–	–	2	–	–	–	–	–	2

16. Feeding other than liquids^6^	–	–	4	–	–	–	–	–	2

17. Bringing liquids to mouth	–	–	4	–	–	–	–	–	3

18. Hygiene	–	–	4	–	–	–	–	–	4

19. Dressing	–	–	4	–	–	–	–	–	3

20. Writing	–	–	4	–	–	–	–	–	3

21. Working^7^	–	–	4	–	–	–	–	–	3

Total	**16**	**16**	**30**	**–**	**–**	**–**	**9**	**13**	**23**

Overall Total Score			**82**						**58**


^1^Definitions for 1–9: 0 = none; 1 = slight; 2 = moderate amplitude; 3 – marked amplitude; 4 = severe amplitude; ^2^Definitons for 10: 0 = normal; 1 = mildly abnormal; 2 = moderately abnormal; 3 = marked abnormal; 4 = severely abnormal; ^3^Definitions for 11–13: 0 = normal; 1 = slightly tremulous; 2 = moderately tremulous; 3 = accomplishes the task with great difficulty; 4 = unable to complete; ^4^Pouring: 0 = normal; 1 = more careful than a person without tremor; 2 = spills a small amount; 3 = spills a considerable amount; 4 = unable to pour water; ^5^Speaking: 0 = normal; 1 = mild voice tremulousness when “nervous” only; 2 = mild voice tremor, constant; 3 = moderate; 4 = severe, some words are difficult to understand; ^6^Definition 16–20: 0 = normal 1 = mildly normal; 2 = moderately abnormal; 3 = markedly abnormal; 4 = severely abnormal; ^7^Working: 0 = tremor does not interfere with job; 1 = able to work, but needs to be more careful than the average person; 2 = able to do everything, but with errors; 3 = unable to do regular job. May have changed to a different job because of tremor; 4 = Unable to do any outside job.

## Discussion

Holmes tremor is commonly a debilitating condition, causing a complex large amplitude and low frequency rest and action tremor, markedly worsened with posture. HT has been previously described during the acute or recovery phase of PML [5,6]. As with the reported patient, while other symptoms can resolve, the patients who survive can present with significant tremor commonly refractory to medical management [5]. Due to the complexity of mechanisms involving Holmes Tremor and variability in clinical response to treatment, there is currently no consensus for its management [7].

Although data is limited, DBS has been shown to provide more robust improvement of overall HT symptoms when compared to medical management [1,8]. One of the largest studies looking at DBS effects on Holmes tremor showed an average tremor improvement of 64% [9]. The thalamic ventral intermediate nucleus (VIM) is the most common target used for HT [10]. Although the subthalamic nucleus is another common target for tremor control, the globus pallidus internus (GPi) can sometimes be used in cases with significant chorea or when the thalamus is severely damaged [2]. Our patient did not demonstrate significant thalamic damage on his MRI, and the VIM/PSA target was chosen.

DBS is reported to be especially more effective in the postural aspect of HT. In our reported case, we demonstrate at least a 2-point improvement on resting, action, and postural tremor on the FTM scale. As was described in a previous case report, it is possible that the PSA stimulation has contributed to better controlling the resting tremor component in our patient [11]. The use of an expanded target area has been theorized to provide a more significant reduction of symptoms in complex tremors such as HT based on a possible pathophysiology that includes not only cerebello- thalamic, but also pallido-thalamic pathways [2].

The improvement of the action tremor continues to be a challenge with both DBS and medication management [7]. Although our patient presented with near resolution of his resting tremor, the associated ataxia continued to significantly impact his function, and was the main symptom responsible for the still moderately elevated scoring of the FTM rating scale with the DBS turned on. The same limitation of intentional and proximal tremor control with the contribution of ataxia was reported for patients in which different targets, including GPi, were used [10]. The caudal zona incerta may be a potential target for improvement of ataxia, but the small quantity of reports avaiable in the literature limits its use [10].

Overall, when compared to patients with essential tremor, patients with HT are found to require higher settings (amplitude, frequency, and pulse width) to achieve tremor improvement [10]. The most common side effects reported in patients with VIM DBS include paresthesias, dysarthria, gait instability and ataxia [12]. With our patient receiving bilateral stimulation, possible side effects were a clear concern. However, as demonstrated in the FTM scale, with appropriate adjustments, his dysarthria was largely unchanged with stimulation. Although his ataxia was not improved, there was no reported worsening. Cognitive decline was also a concern in our reported case. However, PML and chronic HIV are associated with cognitive decline, and the reported mild decrease in executive function one year after his first procedure might be explained by the natural evolution of his underlying disease pathology.

The evaluation of the effects in the case reported is limited by the short time of follow-up from the second DBS implantation. As with the effects of oral medications, concerns of recurrence of tremors overtime when using VIM as a target have been reported. However, at 12 months follow-up from his second surgery, the effects from his first DBS implantation remained noticeable at 24 months post procedure. To our knowledge, this is the first report of bilateral DBS successfully implemented in a patient with Holmes tremor with no significant side effects.
